# Supplementary material for: Effectiveness of Vestibular Rehabilitation after Concussion: A Systematic Review of Randomised Controlled Trial
Source: Healthcare (Basel). 2022 Dec 28;11(1):90. doi: 10.3390/healthcare11010090 (PMC9819464; doi:10.3390/healthcare11010090)
Supplement: Supplementary file 1 [file healthcare-11-00090-s001.zip › healthcare-2135197-supplementary.pdf]

PUBMED

Concussion[MeSH Terms] OR Sport Related Concussion OR Brain Concussion OR mild Traumatic Brain Injury OR Traumatic Brain Injuries AND Vestibular Rehab AND Physical therapy modalities[Mesh] OR "exercise therapy"[Mesh] OR Exercise[Mesh] OR Rehabilitation[Mesh] OR Movement[Mesh] OR "Vestibular Function Tests"[Mesh] OR rehabilitation OR Physiotherap OR (Physical and Therap) OR Habituat OR Exercis OR Epley OR Canalith OR Treatment OR Intervention OR Manoeuvre OR Maneuver OR (Balance and Exercis) OR (Balance and Train) OR "Postural Control" OR Train OR (Sensory and Relearn) OR (Sensory and Retrain) OR (Postural and Relearn) OR (Postural and Retrain) OR Reposition OR (Functional and Retrain*) OR (Visual and Vestibular) OR (Cooksey and Cawthorne) AND ("vertigo"[MeSH Terms] OR "dizziness"[MeSH Terms]) AND (Balance).

WEB of SCIENCE

(Concussion[MeSH Terms] OR Sport Related Concussion OR Brain Concussion OR mild Traumatic Brain Injury OR Traumatic Brain Injuries) *AND* **TOPIC:** (vestibular rehabilitation) *AND* **TOPIC:** (Physical therapy modalities[Mesh] OR "exercise therapy"[Mesh] OR Exercise[Mesh] OR Rehabilitation[Mesh] OR Movement[Mesh] OR "Vestibular Function Tests"[Mesh] OR rehabilitation OR Physiotherap OR (Physical and Therap) OR Habituat OR Exercis OR Epley OR Canalith OR Treatment OR Intervention OR Manoeuvre OR Maneuver OR (Balance and Exercis) OR (Balance and Train) OR "Postural Control" OR Train OR (Sensory and Relearn) OR (Sensory and Retrain) OR (Postural and Relearn) OR (Postural and Retrain) OR Reposition OR (Functional and Retrain*) OR (Visual and Vestibular) OR (Cooksey and Cawthorne)) *AND* **TOPIC:** (vertigo or dizziness or balance)

Cochrane trial

Concussion OR Sport Related Concussion OR Brain Concussion OR mild Traumatic Brain Injury OR Traumatic Brain Injuries in Title Abstract Keyword AND Physical therapy modalities OR exercise therapy OR Exercise OR Rehabilitation OR Movement OR Vestibular Function Tests OR rehabilitation OR Physiotherap OR (Physical and Therap) OR Habituat OR Exercis OR Epley OR Canalith OR Treatment OR Intervention OR Manoeuvre OR Maneuver OR (Balance and Exercis) OR (Balance and Train) OR "Postural Control" OR Train OR (Sensory and Relearn) OR (Sensory and Retrain) OR (Postural and Relearn) OR (Postural and Retrain) OR Reposition OR (Functional and Retrain) OR (Visual and Vestibular) OR (Cooksey and Cawthorne)

CINHAIL and SPortDiscuss

"(Concussion OR Sport Related Concussion OR Brain Concussion OR mild Traumatic Brain Injury OR Traumatic Brain Injuries) AND vestibular rehabilitation AND (Physical therapy modalities OR exercise therapy OR Exercise OR Rehabilitation OR Movement OR Vestibular Function Tests OR rehabilitation OR Physiotherap OR (Physical and Therap) OR Habituat OR Exercis OR Epley OR Canalith OR Treatment OR Intervention OR Manoeuvre OR Maneuver OR (Balance and Exercis) OR (Balance and Train) OR "Postural

Control" OR Train OR (Sensory and Relearn) OR (Sensory and Retrain) OR (Postural and Relearn) OR (Postural and Retrain) OR Reposition OR (Functional and Retrain*) OR (Visual and Vestibular) OR (Cooksey and Cawthorne)) AND (vertigo OR dizziness) AND Balance

PEDro

Concussion

Vestibular rehabilitation
